# Re-entry catheter-guided in situ fenestration to preserve the left subclavian artery during thoracic endovascular aortic repair for subacute type B aortic dissection

**DOI:** 10.1016/j.jvscit.2025.101905

**Published:** 2025-07-09

**Authors:** Diego Ardiles, Marcelo Lagos, Jeison Peñuela, Allan Vera, Rocío Castro, Manuel Espíndola

**Affiliations:** a Cirugía Vascular y Endovascular, Redsalud Mayor, Temuco, Chile; b Cirugía Vascular, Servicio Subespecialidades, Hospital Complejo Asistencial Padre Las Casas, Temuco, Chile; c Cirugía Vascular y Endovascular, Hospital San Juan de Dios, Santiago, Chile; d Residencia Radiología, Universidad de Los Andes, Santiago, Chile; e Servicio Cirugía Vascular, Hospital DIPRECA, Santiago, Chile

**Keywords:** Aortic dissection, Endovascular procedures, Re-entry catheter, In situ fenestration, TEVAR

## Abstract

We describe the use of the BeBack re-entry catheter as an alternative tool for in situ fenestration during thoracic endovascular aortic repair in a patient with subacute type B aortic dissection requiring zone 2 landing. A 69-year-old woman with high-risk imaging features underwent thoracic endovascular aortic repair with intentional coverage of the left subclavian artery. Via percutaneous left brachial access, a deflectable 7F introducer was positioned against the outer curvature of the thoracic endograft. Retrograde puncture was performed using a 4F × 120 cm BeBack re-entry catheter under angiographic guidance in orthogonal projections to ensure precise orientation and penetration. After successful crossing of the endograft fabric, an 0.018″ guidewire was advanced into the ascending aorta, followed by sequential dilation and deployment of a balloon-expandable stent. Final angiography confirmed patency of the target vessel and exclusion of the false lumen.

Preservation of supra-aortic branches during thoracic endovascular aortic repair (TEVAR) remains a technical challenge, particularly when the left subclavian artery must be covered. This case demonstrates the potential usefulness of re-entry catheter-assisted in situ fenestration (ISF) as an alternative to laser-based techniques, which may not be available in all centers. The approach enabled successful revascularization of the subclavian artery without the need for surgical bypass. The use of a directional re-entry device offers greater control during puncture and may expand the applicability of ISF in both emergent and elective settings, improving patient outcomes and procedural flexibility.

Endovascular treatment of type B aortic dissection has progressed significantly, allowing for less invasive interventions with favorable clinical outcomes. However, coverage of supra-aortic branches during endovascular repair is common and poses important technical challenges. The need to maintain cerebral and spinal cord perfusion has driven the development of techniques such as ISF, using tools such as lasers, needles, or specialized re-entry devices.^1^, ^2^, ^3^, ^4^, ^5^, ^6^ Although lasers are among the most widely studied tools for ISF,^3^^,^^5^ their limited availability in many centers has stimulated interest in alternative methods. Among them, devices such as the BeBack catheter (Bentley, Hechingen, Germany) have emerged as an effective solution, enabling controlled and directional punctures in thoracic endografts.^7^^,^^8^ This technique provides a safe and reproducible option to preserve supra-aortic branches in complex scenarios, even outside of emergency settings.^2^^,^^4^^,^^6^

We present a case of subacute type B aortic dissection treated with TEVAR landing in zone 2 with ISF using the BeBack re-entry catheter. Written informed consent was obtained from the patient for publication of this case and accompanying images.

## Case and technique

A 69-year-old woman with subacute type B aortic dissection exhibiting high-risk clinical features of hypertension and refractory chest pain and imaging findings of a 46-mm descending aortic diameter, a 27-mm false lumen, and a 15-mm primary entry tear underwent TEVAR under general anesthesia. The patient had a history of heavy smoking, chronic obstructive pulmonary disease, and coronary artery disease. Given the high risk of requiring two separate surgical procedures or a prolonged operation to optimize the proximal landing zone, open repair was deemed to have an excessively high risk. Therefore, we opted for a single-stage, minimally invasive endovascular TEVAR with ISF. A left femoral venous puncture was performed to place a temporary pacemaker lead in the right ventricle. Via percutaneous right femoral artery access, using a preclosure technique, two Prostyle devices (Abbott, Chicago, IL) were deployed to secure access with a 10F × 10 cm introducer. Under intravascular ultrasound guidance using the Visions PV 0.035″ system (Philips, Amsterdam, the Netherlands), a stiff hydrophilic 0.035" guidewire (Terumo, Tokyo, Japan) was advanced through the true lumen into the ascending aorta. This was exchanged for a Lunderquist support guidewire (Cook Medical, Bloomington, IN), followed by progressive dilation with 2F stepwise dilators (Cook Medical) and insertion of a 20F DrySeal introducer (W. L. Gore & Associates, Flagstaff, AZ).

Left brachial percutaneous access was obtained with a 5F × 25 cm radial introducer (Terumo), through which a 5F centimeter-marked pigtail catheter (Cook Medical) was positioned in the ascending aorta.

**Fig 1:**
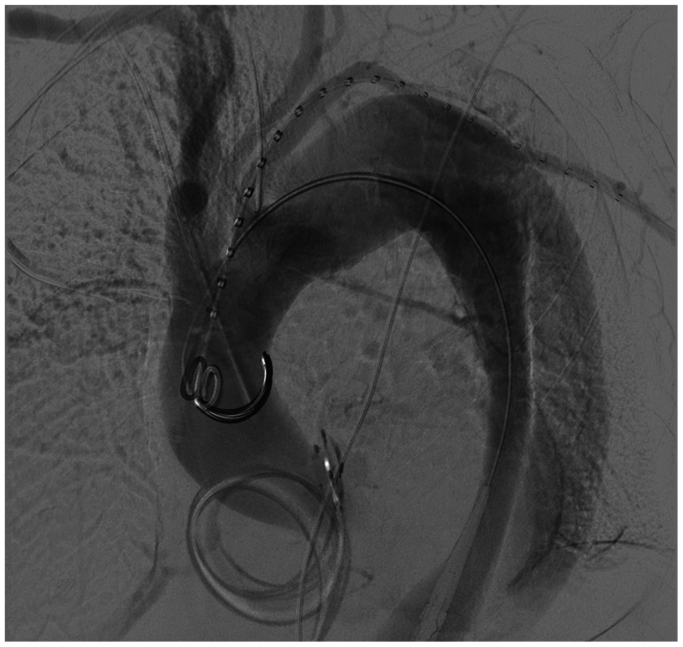
Initial aortography in left anterior oblique (LAO) projection showing the dissection site and landing zone in zone 2, along with anatomical landmarks for stent deployment in the left subclavian artery.

Via right femoral access, a Relay Pro NBS 30-26-200 endograft (Terumo) was advanced. Angiographic projection and reference aortography were performed from the pigtail catheter, visualizing the origin of the left subclavian artery, internal mammary artery, and vertebral artery (Fig 1). Rapid pacing was initiated, decreasing the mean arterial pressure to 55 mm Hg, followed by deployment of the thoracic endograft as planned, extending over the bovine trunk and covering zone 2. Deployment was performed with the nose cone held in a fixed, unreleased position to preserve curvature control and prevent deformation of the graft during puncture and balloon crossing, facilitating ISF and avoiding the use of a Coda balloon, as described in the literature, owing to its association with retrograde aortic dissection.

**Fig 2:**
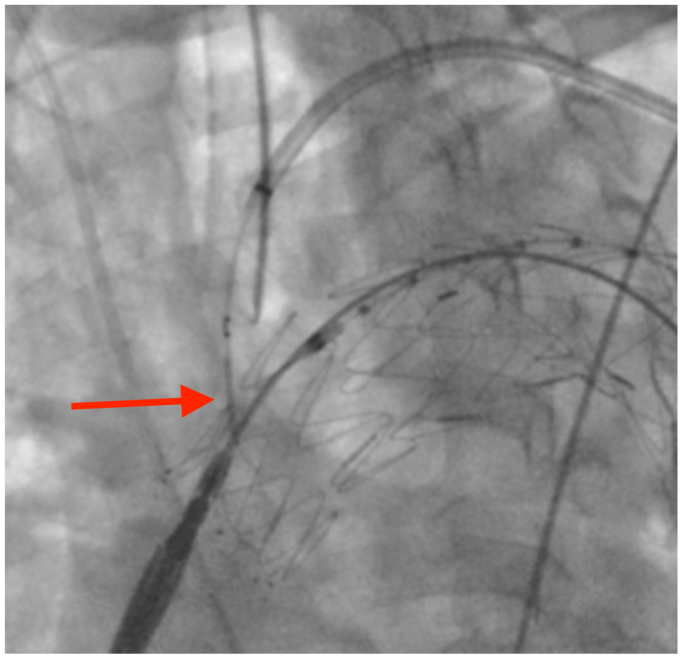
Puncture of the outer curvature of the endograft using a BeBack 4F × 120 cm system. The nose cone of the RelayPro NBS endograft is maintained in place to provide support during puncture and facilitate device passage through the fenestration, avoiding the need for a Coda balloon in this area and preserving the endograft's conformation without deformation during force application. *Red arrow* indicate the exact puncture site with the BeBack system on the greater curvature of the endograft.

The radial introducer was then exchanged for a deflectable Braidin MG 7F × 83 cm introducer (APT Medical, Shenzhen, China). Pullback and deflection were performed by pressing the introducer tip against the endograft. Through a 9F femoral introducer on the left side, intravascular ultrasound examination was used to visualize the entry and passage of the guidewire within the endograft; however, it did not allow adequate visualization, limiting its usefulness. From the left brachial access in a retrograde direction, a BeBack re-entry catheter 4F × 120 cm was advanced. Positioning was confirmed in two orthogonal angiographic projections as we carefully guided the BeBack catheter through a deflectable sheath, ensuring that the puncture remained between stent struts and allowing controlled needle advancement for direct graft puncture (Fig 2).

**Fig 3:**
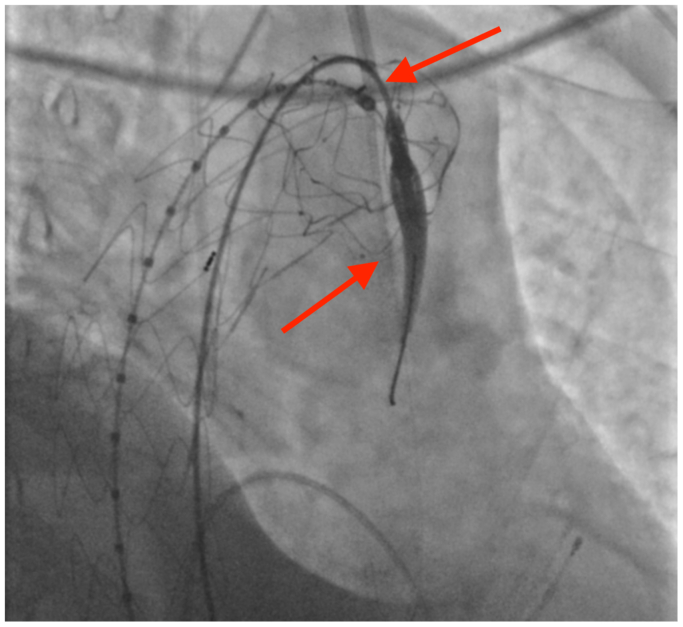
Confirmation of intragraft positioning in right anterior oblique (RAO) projection. *Red arrows* indicate the puncture site and 0.018″ guidewire passage. The same RAO projection was used before puncture to verify BeBack catheter apposition between stent struts and at the dome of the prosthesis for secure fabric perforation, emphasizing the critical role of double projection for procedural success and safety.

**Fig 4:**
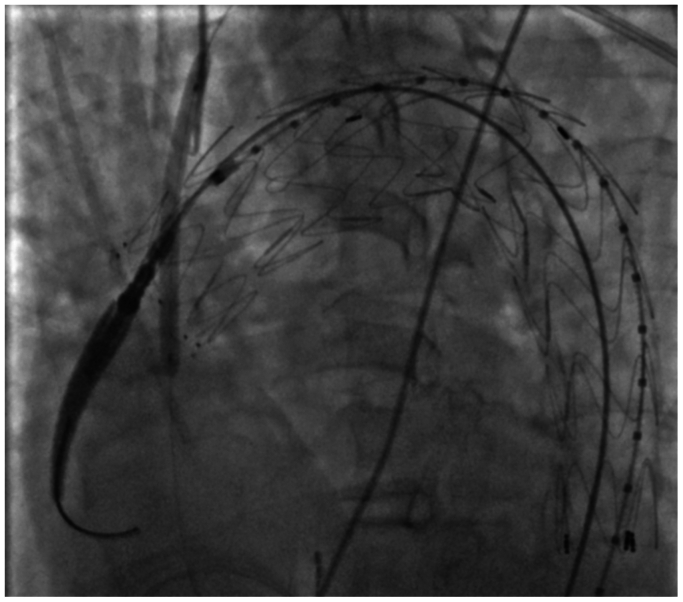
Sequential dilation using Armada 018 balloons, starting with 2 mm, followed by 3, 4, and 6 mm. Progressive dilation is key to avoiding fabric tears in the endograft.

After puncture, an A V18 guidewire (Boston Scientific, Marlborough, MA) was introduced through the BeBack system into the ascending aorta and its position was confirmed in two planes (Fig 3). Over this wire, a Rubicon 0.018″ crossing catheter (Boston Scientific) was used to widen the puncture, followed by stepwise dilation with Armada 0.018" balloons (Abbott) of 2, 3, 4, and 6 mm (Fig 4). A 125-cm vertebral catheter was used to exchange for an Amplatz Super Stiff support wire (Boston Scientific) placed into the ascending aorta.

**Fig 5:**
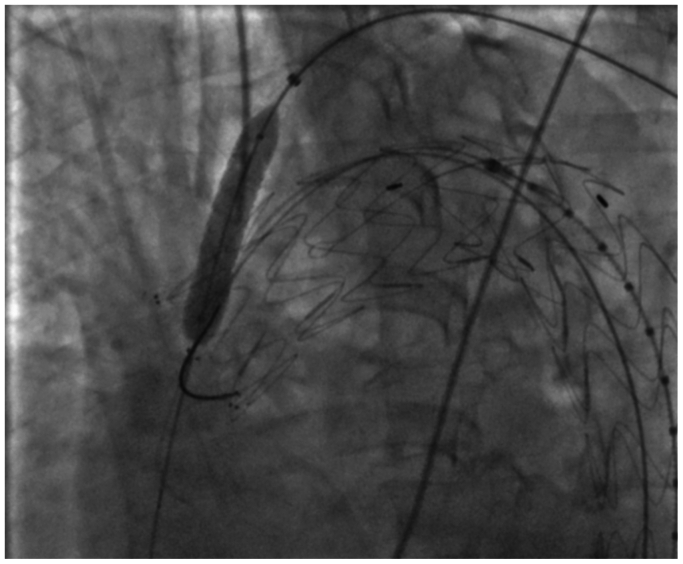
Deployment of an 8 × 37 mm BeGraft stent. The stent is deployed using a pullback technique of the introducer sheath once the stent has been advanced beyond it to the target area. A safety margin of 8 mm was maintained because the endograft fabric demonstrated erratic behavior beyond 10 mm during bench testing.

**Fig 6:**
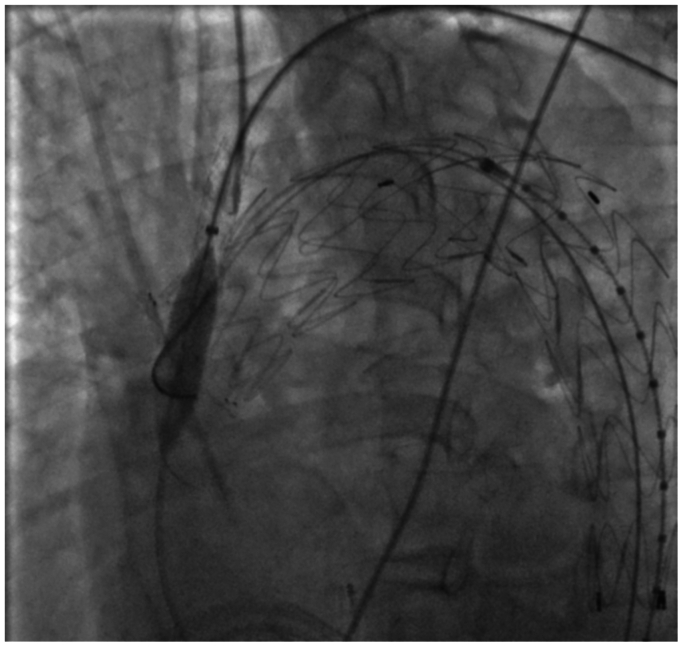
Proximal flaring of the stent graft using a 9 × 20 mm Mustang balloon to ensure proper sealing and prevent migration. The maneuver is repeated distally, matching the stent 1:1 with the subclavian artery and achieving a 20-mm landing zone. The crossing zone of the endograft was respected throughout the procedure.

Final deployment of the main endograft was completed. The deflectable introducer was replaced with a Prelude Roadster 7F × 70 cm introducer (Merit Medical, South Jordan, UT), and the fenestration was crossed to deploy a Begraft stent 8-37 mm (Bentley), with proximal flaring using a Mustang 9-20 mm balloon (Boston Scientific) and distal postdilation to match the subclavian artery diameter using the same balloon (Figs 5 and 6). This series of maneuvers—from fenestration with the BeBack catheter through serial Armada balloon dilations and distal postdilation to match the subclavian artery diameter—was completed in approximately 40 minutes.

**Fig 7:**
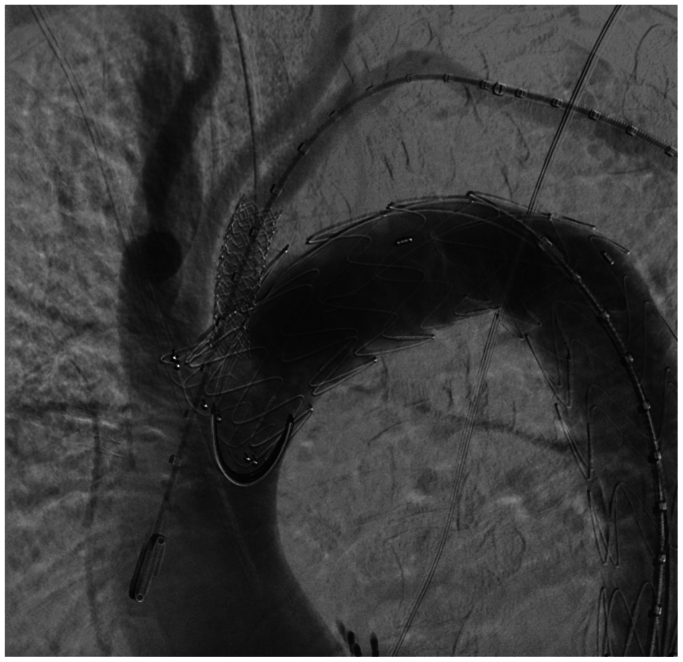
Final angiographic control of the in situ fenestration (ISF) site. The stent graft was positioned properly, maintaining patency of the left subclavian artery with no opacification of the false lumen.

Finally, the dissection was treated with a Relay Pro NBS 28-24-150 extension (Terumo) down to the upper edge of the celiac trunk, and a PETTICOAT technique was applied using a 36-120 dissection stent (Cook Medical) into the infrarenal segment, as is routine in our institution. Final control showed satisfactory results with preserved left subclavian artery patency (Fig 7).

## Discussion

ISF has emerged as an effective strategy to preserve supra-aortic branches during complex thoracic endovascular repairs. Although laser is used widely and has shown favorable outcomes,^2^^,^^3^^,^^5^ its cost and limited availability represent important constraints.

To further underscore its practical benefits, we performed bench tests simulating sequential balloon dilations after BeBack fenestration. The endograft fabric tolerated expansions up to 10 mm without tearing, and—crucially—the BeBack catheter allows immediate passage of a 0.018″ guidewire through the newly created fenestration. These findings suggest that sequential dilation after fabric puncture is safe for creating a secure opening and facilitating prompt cannulation, potentially improving procedural efficiency and reliability compared with laser-based techniques.

In this context, needle-based techniques and re-entry devices such as the BeBack catheter offer a viable alternative.^1^^,^^2^^,^^4^^,^^8^^,^^9^ The device allows precise and stable puncture of the endograft, with a manageable learning curve and low complication profile, as reported in recent experiences.^7^^,^^8^ Compared with other tools, its directional control facilitates accurate retrograde fenestration from brachial access, as demonstrated in this case. This factor can obviate the need for adjunctive procedures such as carotid-subclavian bypass, potentially reducing the risk of spinal cord ischemia.^6^

Moreover, the adaptation of ISF techniques using devices originally designed for other clinical contexts—such as peripheral arterial occlusions—enables the extension of endovascular indications even in nonemergent settings, as seen in this subacute dissection case, offering promising clinical outcomes.^6^^,^^8^

## Conclusions

The use of the BeBack device is a feasible and effective alternative for ISF of thoracic endografts. This technique enables rapid and clean access, allowing preservation of the subclavian artery without the need for a carotid-subclavian bypass. This may reduce the risk of spinal cord ischemia and technically simplify the procedure in selected patients.

## Declaration of generative Ai and Ai-assisted technologies in the writing process

Artificial intelligence tools (ChatGPT, OpenAI, March 2025 version) were used in the preparation of the English language and structure of the abstract and supporting text. The authors reviewed and edited the final version to ensure accuracy and appropriateness.

## Funding

None.

## Disclosures

None.
